# Mid-Pregnancy Maternal Anxiety Mediates the Association Between Maternal Chronotype and Breastfeeding Duration

**DOI:** 10.3390/nu18030405

**Published:** 2026-01-26

**Authors:** Nur K. Abdul Jafar, Elaine K. H. Tham, Doris Fok, Mei Chien Chua, Oon-Hoe Teoh, Daniel Y. T. Goh, Lynette Pei-Chi Shek, Fabian Yap, Kok Hian Tan, Peter D. Gluckman, Yap-Seng Chong, Michael J. Meaney, Birit F. P. Broekman, Wei Wei Pang, Shirong Cai

**Affiliations:** 1 Institute for Human Development and Potential (IHDP), Agency for Science, Technology and Research (A*STAR), Singapore 117609, Singapore; nur.abduljafar@monash.edu (N.K.A.J.);; 2 Department of Neonatology, Khoo Teck Puat, National University Hospital, Singapore 119074, Singapore; 3 Department of Neonatology, Kandang Kerbau Women’s and Children’s Hospital, Singapore 229899, Singapore; 4 Department of Paediatrics, Kandang Kerbau Women’s and Children’s Hospital, Singapore 229899, Singapore; 5 Department of Paediatrics, Yong Loo Lin School of Medicine, National University of Singapore, Singapore 119228, Singapore; 6 Department of Maternal Fetal Medicine, Kandang Kerbau Women’s and Children’s Hospital, Singapore 229899, Singapore; 7 Liggins Institute, University of Auckland, Auckland 1023, New Zealand; 8 Department of Obstetrics & Gynaecology, Yong Loo Lin School of Medicine, National University of Singapore, Singapore 119228, Singapore; 9 Department of Psychiatry and Neurology and Neurosurgery, McGill University, Montreal, QC H4A 3J1, Canada; 10 Department of Psychiatry, OLVG and Amsterdam UMC, VU University, 1081 HV Amsterdam, The Netherlands; 11 Departments of Global Centre for Asian Women’s Health, Dean’s Office and Bia-Echo Asia Centre for Reproductive Longevity and Equality, Yong Loo Lin School of Medicine, National University of Singapore, Singapore 119228, Singapore; 12 Human Potential Translational Research Programme, Yong Loo Lin School of Medicine, National University of Singapore, Singapore 117597, Singapore

**Keywords:** chronotype, sleep, breastfeeding duration, depression, anxiety, mid-pregnancy

## Abstract

**Background:** Maternal chronotype, maternal sleep, and breastfeeding practices are separately associated with maternal mood. However, it is not known if maternal mood mediates the associations between maternal chronotype or maternal sleep and breastfeeding duration. **Objective:** To investigate whether maternal mood mediates the associations of maternal chronotype and maternal prenatal sleep with breastfeeding duration in a multiethnic cohort of Singaporean mothers. **Methods:** In a prospective cohort study, caregivers of term-born, singleton infants (*N* = 340) completed the Horne–Östberg Morningness–Eveningness Questionnaire (MEQ; 54 months), Pittsburgh Sleep Quality Index (PSQI; 26 weeks gestation), Edinburgh Postnatal Depression Scale (EPDS; 26–28 weeks gestation) and State–Trait Anxiety Inventory (STAI-state, STAI-trait; 26–28 weeks gestation) and reported breastfeeding practices from 3 weeks to ≥12 months. Regression and mediation analyses were adjusted for maternal age, parity, maternal education, ethnicity, pre-pregnancy BMI, and mode of delivery. **Results:** Morningness was significantly associated with a longer breastfeeding duration (*β* = 0.02, *p* = 0.037) and lower maternal anxiety symptoms (STAI-state: *β* = −0.19, *p* = 0.006 and STAI-trait: *β* = −0.18, *p* = 0.004). Lower maternal anxiety symptoms were significantly associated with a longer breastfeeding duration (STAI-state: *β* = −0.02, *p* = 0.003; STAI-trait: *β* = −0.02, *p* = 0.016). STAI-state, but not STAI-trait or EPDS, mediates the association between maternal chronotype and breastfeeding duration (*β*_indirect_ = 0.004 (0.0004, 0.009)). Maternal mood did not mediate the association between maternal night sleep duration and breastfeeding duration. **Conclusions:** Maternal state-anxiety constitutes a behavioral pathway through which maternal chronotype influences breastfeeding duration. Strategies to target maternal anxiety in pregnant women with eveningness tendencies to promote breastfeeding duration are warranted.

## 1. Introduction

It is well-known that breastfeeding benefits both mother and child [1], but the maintenance of breastfeeding rates remains below WHO recommendations [2,3,4,5,6]. Many factors contribute to breastfeeding success [7,8], including genetics, diet, environment, social factors, demographics, and behavioral factors [5,8,9,10]. The literature has well established a bidirectional link between maternal psychological distress during pregnancy and/or the postpartum period and breastfeeding [7,11,12,13]. It has been proposed that maternal psychological distress may decrease milk production via impairing oxytocin release, elevating cortisol levels, and decreasing insulin sensitivity [7]. A review by Hoff et al. (2019) revealed negative associations between postpartum anxiety and breastfeeding initiation, duration, and exclusivity [13]. Unfortunately, the limited evidence is mixed regarding the association between prenatal anxiety and breastfeeding duration [13,14]. While some studies found associations between high prenatal anxiety and a greater risk of early cessation of breastfeeding [15,16,17], others did not find any associations [14,18,19]. Dias et al. (2015) reviewed studies showing that antenatal and postpartum depression were associated with shorter breastfeeding durations [12]. In addition, breastfeeding practices may mediate the association between depression during pregnancy and postpartum [12]. A recent review further highlights the impact of maternal psychological distress during the pregnancy and postpartum periods on poor breastfeeding outcomes, such as a shortened duration of breastfeeding [7].

Although much research has demonstrated associations between evening chronotype and poor mental health outcomes [20,21], few studies have examined this link among pregnant women [22,23,24,25]. One longitudinal study found that pregnant women with an evening chronotype had a higher risk of developing perinatal depressive symptoms in the first month after delivery [22]. In contrast, a cross-sectional study found no significant associations between chronotype and symptoms of depression and anxiety at different perinatal timepoints from late pregnancy to two years postpartum [25]. Chronotype is considered a relatively stable trait in adults aged 18–65 years [26]. Although some studies have reported temporary shifts in chronotype during pregnancy, such as an earlier chronotype in the first and second trimesters that returns to the pre-pregnancy state by the third trimester [27,28], these findings support its conceptualization as a trait-like characteristic suitable for examining longer-term associations with maternal and child outcomes.

During the perinatal period, reduction in sleep duration and/or quality, as well as increased mood disturbances, are common in women [29,30]. Improved perinatal mood disorders may be the result of a gradual reduction in night-time sleep disturbances in pregnant women, but research in this area is still developing [30]. While some studies reported that women who breastfeed had a longer night sleep duration during the postpartum period [31,32], only one study reported that greater hours of night sleep during the postpartum period were significantly associated with a longer duration of breastfeeding [33]. Hence, it remains unclear whether maternal sleep duration during the perinatal period influences breastfeeding duration and if maternal mood influences this association.

To the best of our knowledge, the mediating role of maternal mood on the associations between maternal chronotype, maternal prenatal sleep, and breastfeeding duration has not been previously studied. This study is the first to examine these relationships in a multiethnic Asian cohort, providing novel insights into how maternal circadian preference and prenatal mood may influence breastfeeding behaviors. The present study of Singaporean mothers, therefore, aimed to investigate the associations between maternal chronotype, prenatal night sleep duration and breastfeeding duration, and whether these associations are mediated by maternal prenatal mood. We postulated that maternal mood mediates the associations between maternal chronotype or prenatal night sleep duration and breastfeeding duration. Specifically, we hypothesized that mothers with greater morningness tendencies or longer prenatal night sleep duration would be more likely to breastfeed for a longer duration, and that these associations would be statistically mediated by lower levels of maternal depressive symptoms and anxiety during mid-pregnancy. A systematic review suggested that state-anxiety is more likely to impact breastfeeding duration than trait-anxiety [13]; therefore, we hypothesized that current anxiety (state-anxiety) would be more strongly associated with breastfeeding duration, and thus more likely to mediate these associations, than long-term anxiety (trait-anxiety).

## 2. Materials and Methods

### 2.1. Study Design and Sample

**Figure 1:**
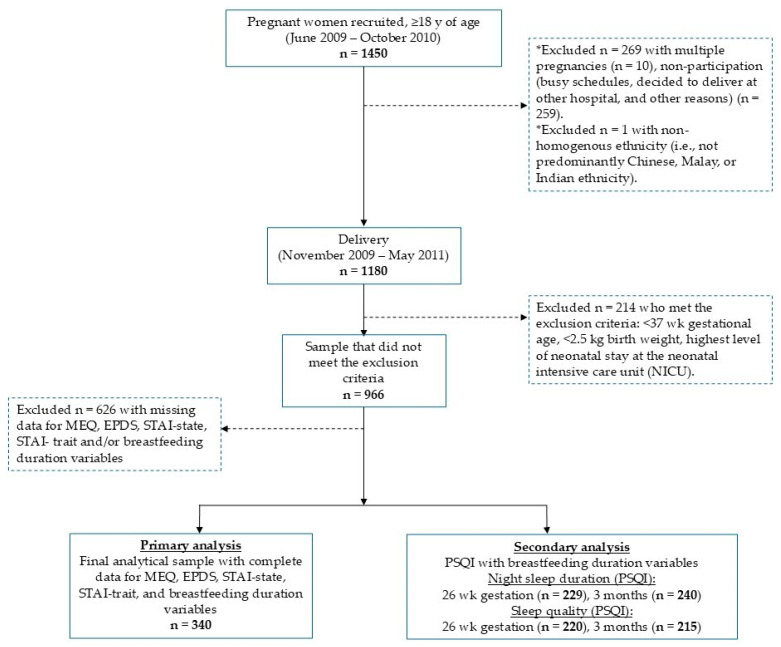
Study flow chart. Flowchart describing the data available for MEQ, PSQI, EPDS, STAI-state, STAI-trait, and breastfeeding duration. Thick solid arrows indicate the distribution of participants at each stage of the study and dotted arrows represent participants who were excluded from this study. EPDS, Edinburgh Postnatal Depression Scale; MEQ, Morningness–Eveningness Questionnaire; PSQI, Pittsburgh Sleep Quality Index; STAI, State–Trait Anxiety Inventory. * Excluded participants included those with multiple pregnancies, non-participation, or non-homogenous ethnicity.

*N* = 1450 pregnant women in the first trimester, aged ≥ 18 years, were recruited from the National University Hospital and Kandang Kerbau Women’s and Children’s Hospital in Singapore between June 2009 and October 2010 to participate in the GUSTO (Growing Up in Singapore Towards healthy Outcomes) birth cohort study [34]. In this study, the infants were born between November 2009 and May 2011. In the current study, we included 966 mother–offspring pairs and excluded those who met the following exclusion criteria (*N* = 269): multiple pregnancies, gestational age < 37 weeks, birth weight < 2.5 kg, and highest level of neonatal stay at the neonatal intensive care unit (NICU) (Figure 1). Non-participation at this period (*N* = 259) was due to reasons such as mothers deciding to deliver at other hospitals or busy schedules. Out of the 966 participants included in this study, a total of 340 (35.2%) participants completed measures on all study questionnaires for maternal chronotype (i.e., MEQ), maternal mood (i.e., EPDS, STAI-state, and STAI-trait), and breastfeeding practices (Figure 1). Out of 340 participants, the maternal night sleep duration measure from the PSQI questionnaire was completed at 26–28 weeks of gestation and most had breastfeeding duration data recorded (*N* = 229) (Figure 1).

This study was approved by both the National Health Care Group Domain Specific Review Board and the SingHealth Centralized Institutional Review Board. All participants who provided data gave informed written consent before their participation (DSRB D/2009/00021, 26 February 2009; CIRB 2018/2767/D, 2 March 2009).

### 2.2. Measures

#### 2.2.1. Maternal Chronotype

Participants completed a 19-item Horne-Östberg Morningness–Eveningness Questionnaire (MEQ) to assess their individual diurnal preference, with total scores ranging from 16 to 86 [35]. Higher MEQ scores represent those participants with greater morningness tendencies, while lower MEQ scores represent those participants with greater eveningness tendencies [35]. In this study, mothers were administered the MEQ at 54 months postpartum, and MEQ scores were analyzed in a continuous manner.

#### 2.2.2. Maternal Sleep Duration

- Pittsburgh Sleep Quality Index (PSQI)

The PSQI has 7 measures of sleep quality: subjective sleep quality, sleep latency, sleep duration, habitual sleep efficiency, sleep disturbances, use of sleeping medication, and daytime dysfunction [36]. Night sleep duration was determined from the question: “*During the past month, how many hours of actual sleep did you get at night?*” In this study, mothers were administered the PSQI at 26–28 weeks of gestation.

#### 2.2.3. Maternal Mood

- Edinburgh Postnatal Depression Scale (EPDS)

Maternal depressive symptoms during the mid-pregnancy period were assessed using the EPDS [37], which has been validated in Singaporean women [38]. The EPDS is a self-report instrument that contains 10 items of common depressive symptoms over the past week, with total scores ranging from 0 to 30. In this study, mothers were administered the EPDS at 26–28 weeks of gestation, and EPDS scores were analyzed in a continuous manner.

- State–Trait Anxiety Inventory (STAI)

Maternal anxiety symptoms during the mid-pregnancy period were assessed using the Spielberger State–Trait Anxiety Inventory [39]. The STAI consists of 40 items with a 4-point Likert scale assessing both transient (“STAI-state” measure) and stable (“STAI-trait” measure) characteristics of anxiety. The scores for the STAI were reverse-scored and summed. In this study, mothers were administered the STAI at 26–28 weeks of gestation, and all STAI scores were analyzed in a continuous manner.

#### 2.2.4. Breastfeeding Duration

The infant feeding questionnaires were administered by researchers to mothers at 3 weeks and 3, 6, 9, and 12 months postpartum. Mothers were also asked when they stopped breastfeeding and when they introduced solid food to their infants. The breastfeeding initiation rates in our cohort study (>90% across ethnic groups) were high and remained high in the first month; however, full breastfeeding (i.e., intake of breast milk, with or without water) rates were low (<30% across ethnic groups) [5]. At 26–28 weeks of gestation, a high proportion, 324 (95.3%), of participants in our sample declared their intention to breastfeed.

In this study, the duration of any breastfeeding was categorized into 5 groups as follows: <1 month, 1 to <3 months, 3 to <6 months, 6 to <12 months, and ≥12 months [40]. These categories were defined a priori to reflect clinically meaningful breastfeeding milestones, including early cessation (<1 month), short-term breastfeeding (<3 months), and durations aligned with international recommendations for breastfeeding at ≥6 months and ≥12 months [1]. Breastfeeding duration was analyzed in a continuous manner to assess the mediation effect of maternal mood on the associations between maternal chronotype or maternal night sleep duration and breastfeeding duration.

### 2.3. Covariates and Other Data

Socioeconomic and demographic data, such as maternal age, maternal ethnicity, maternal education, household income, and parity, were obtained from questionnaires administered to the caregivers by researchers during the antenatal period. Birth and maternal outcomes, such as birth weight, sex of child, gestational age, and mode of delivery, were documented by midwives at delivery. Other maternal variables, such as pre-pregnancy BMI and gestational diabetes mellitus (GDM) status, were also obtained during the antenatal period from medical records and Oral Glucose Tolerance Test (OGTT) results, respectively. Of the 340 mother–offspring pairs with complete data for maternal chronotype, maternal mood, and breastfeeding duration, and included in the final analysis, *N* = 32 had missing data for pre-pregnancy BMI, *N* = 13 for GDM status, *N* = 5 for maternal education, and *N* = 18 for household income. Therefore, for each analysis in this study, the maximum number of mother–offspring pairs that were available was used.

### 2.4. Statistical Analysis

Preliminary analyses were based on one-way analysis of variance (ANOVA) tests with Dunnett’s post hoc multiple comparisons relative to <1 month duration of any breastfeeding group, and Chi-square tests were used to compare the infant and maternal characteristics, maternal chronotype, maternal night sleep duration, and maternal mood variables (i.e., EPDS, STAI-state, and STAI-trait) between breastfeeding duration groups. Chi-square tests with two-sided Fisher’s exact tests, and Mann–Whitney U tests were conducted to identify whether there were any significant differences in participant characteristics between those participants who were included compared to those excluded in the final analysis.

**Figure 2:**
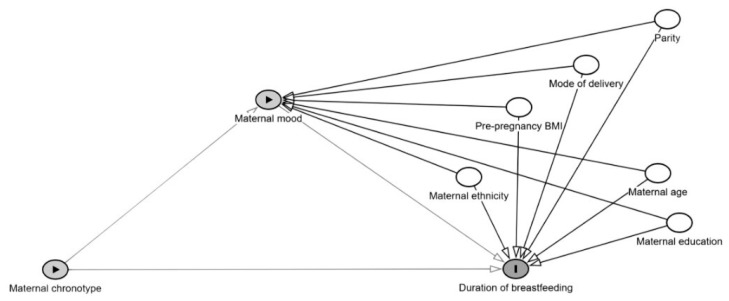
A directed acyclic graph (DAG) illustrating the conceptual mediation model based on Hayes’ PROCESS Model 4. Maternal chronotype is specified as the predictor, maternal mood as the mediator, and duration of breastfeeding as the outcome. The diagram represents both the direct association between maternal chronotype and breastfeeding duration and the indirect association through maternal mood. Maternal age, pre-pregnancy BMI, mode of delivery, maternal ethnicity, parity, and maternal education are included as covariates to account for potential confounding. The model reflects an assumed temporal ordering of variables and adjustment for relevant maternal characteristics. The DAG was generated using DAGitty (https://www.dagitty.net/) (accessed on 18 January 2026).

Using breastfeeding duration as a continuous measure, separate multivariable linear regression analyses with the enter method were conducted to assess the individual associations between maternal chronotype, maternal night sleep duration, or maternal mood (as independent variables) and breastfeeding duration (as the dependent variable) and the associations between maternal chronotype or maternal night sleep duration (as independent variables) and maternal mood variables (as dependent variables). For the mediation analysis, maternal mood variables were tested for mediation on the associations between maternal chronotype or maternal night sleep duration (as independent variables) and breastfeeding duration (as dependent variable) (Figure 2). Mediation analyses were conducted using the mediation model 4 of Hayes’ PROCESS macro on SPSS software version 29 (IBM Corp., Armonk, NY, USA) [41]. The confidence intervals (CIs) were produced using a bias-corrected method with 10,000 bootstrap samples drawn with replacement from the dataset to estimate a sampling distribution for the indirect mediation pathway. The total effect (c-path) refers to the influence of the independent variable (maternal chronotype/night sleep duration) on the dependent variable (breastfeeding duration). The indirect effect refers to the influence of the independent variable (maternal chronotype/night sleep duration) on the dependent variable (breastfeeding duration) through the intervening variable ‘mediator’ (maternal mood). The indirect effect is obtained by multiplying the direct-effect coefficients of the a- and b-paths (i.e., ab). A significant mediation effect occurs when the 95% CIs of the indirect effect do not include zero. Given the observational study design, the mediation analyses were intended to assess statistical associations rather than causal or mechanistic pathways.

Potential confounding factors (maternal age, parity, maternal education, ethnicity, pre-pregnancy BMI, and mode of delivery) included in the analyses were identified from the literature [5,8,9,10]. The regression and mediation analyses were finally adjusted for maternal age, parity, maternal education, ethnicity, and pre-pregnancy BMI in model 1. In addition to these confounding factors, the mode of delivery was also adjusted for in model 2. Maternal chronotype may influence breastfeeding practices as well as mood in women with GDM. Furthermore, sleep impairment can increase the risk of developing GDM [42]. It has been shown that the evening-type chronotype in pregnant women with GDM is associated with depressive symptoms during pregnancy [43]. Women with GDM also tend to breastfeed for a shorter duration [44]. With that, sensitivity analyses were conducted by additionally adjusting for GDM status in the final mediation analyses. Assumptions underlying the statistical analyses were evaluated for all variables. For the adjusted regression analyses, multicollinearity was assessed using collinearity diagnostics, including variance inflation factors (VIFs) for each variable. Collinearity diagnostics showed acceptable VIF (1.06–2.12) and tolerance (0.47–0.95) values, indicating no multicollinearity concerns. The Durbin–Watson statistic of 2.0 indicated independence of residuals. Normality and homoscedasticity assumptions were assessed via visual inspection of normal P–P plots and residual scatterplots and were deemed satisfactory. The Pearson and Deviance goodness-of-fit tests for the adjusted multinomial logistic regression analyses overall suggested a good fit of the data in the models (all *p* > 0.05). Unless otherwise specified, all *p*-values were 2-sided, and the level of significance was considered at *p* < 0.05. Data analyses were conducted using SPSS Version 26 (IBM Corp., Armonk, NY, USA) [45].

## 3. Results

### 3.1. Participant Characteristics

Table 1 shows a comparison of infant and maternal characteristics, maternal chronotype, maternal sleep duration, and maternal mood variables across the duration of any breastfeeding groups. Compared with mothers who breastfed for <1 month, we observed that a greater proportion of mothers who breastfed for 6 to 12 months and/or ≥12 months were more likely to be of Chinese ethnicity (*p* = 0.021 and *p* = 0.19, respectively), have a higher maternal education (i.e., ≥university) (both *p* < 0.001), and higher household income (i.e., ≥$4000) (both *p* < 0.001). Mothers who breastfed longer tended to be older (*p* = 0.021). Likewise, mothers who breastfed for 1 to <3 months had a higher pre-pregnancy BMI compared to those who breastfed for <1 month (*p* = 0.047). Moreover, mothers who breastfed longer tended to have more morningness tendencies, lower state-anxiety and trait-anxiety scores compared to those who breastfed for <1 month (3 to <6 months: *p* = 0.012, *p* = 0.014, and *p* = 0.005, respectively; 6 to 12 months: *p* = 0.002, *p* < 0.001, and *p* < 0.001, respectively; and ≥12 months: *p* = 0.019, *p* < 0.001, and *p* = 0.001, respectively).

No statistically significant differences were seen in the child’s sex, birth weight, gestational age, parity, GDM status, mode of delivery, EPDS score, and maternal night sleep duration (Table 1) across breastfeeding duration groups. No statistically significant differences were observed in the child’s sex, birth weight, gestational age, maternal ethnicity, pre-pregnancy BMI, GDM status, maternal education, household income, and mode of delivery between those participants who were included and those who were not included in the final analysis (see Supplementary Table S1). However, those participants who were included in the final analysis were more likely to comprise nulliparous (*p* < 0.001) and older mothers (*p* = 0.009) (see Supplementary Table S1).

### 3.2. Associations Between Maternal Chronotype, Maternal Mood, and Breastfeeding Duration

After adjusting for potential covariates, our results revealed overall significant associations of MEQ, STAI-state, and STAI-trait scores with breastfeeding duration (Table 2). In models 1 and 2, morningness was significantly associated with a longer duration of any breastfeeding (*F*(1, 301) = 4.07, *p* = 0.045, R^2^ = 0.240; and *F*(1, 300) = 4.38, *p* = 0.037, R^2^ = 0.245, respectively). Moreover, lower STAI-state (*F*(1, 301) = 8.67, *p* = 0.003, R^2^ = 0.251; and *F*(1, 300) = 8.96, *p* = 0.003, R^2^ = 0.257, respectively) and STAI-trait scores (*F*(1, 301) = 6.10, *p* = 0.014, R^2^ = 0.245; and *F*(1, 300) = 5.92, *p* = 0.016, R^2^ = 0.249, respectively) were significantly associated with a longer duration of any breastfeeding. No significant associations between EPDS score and breastfeeding duration were seen in all models (all *p* > 0.05).

After adjusting for potential covariates, our results revealed overall significant associations between MEQ scores and both STAI-state and STAI-trait scores (Table 3). In models 1 and 2, morningness was significantly associated with lower STAI-state (*F*(1, 301) = 7.91, *p* = 0.005, R^2^ = 0.123; and *F*(1, 300) = 7.75, *p* = 0.006, R^2^ = 0.124, respectively) and STAI-trait scores (*F*(1, 301) = 8.48, *p* = 0.004, R^2^ = 0.085; and *F*(1, 300) = 8.64, *p* = 0.004, R^2^ = 0.086, respectively). No significant associations between MEQ score and EPDS score were seen in all models (all *p* > 0.05).

Based on these results, only maternal anxiety (i.e., STAI-state and STAI-trait) was significantly associated with both maternal chronotype and breastfeeding duration.

### 3.3. Associations Among Maternal Sleep Duration, Maternal Mood, and Breastfeeding Duration

Overall, our results revealed no significant association of maternal night sleep duration with breastfeeding duration (Table 2) and all maternal mood variables (Table 3) (all *p* > 0.05).

### 3.4. Maternal Anxiety Mediates the Association Between Maternal Chronotype and Breastfeeding Duration

Table 4 presents the mediation results when maternal mood is the mediator of the association between maternal chronotype and breastfeeding duration. After adjusting for potential covariates, in both models 1 and 2, only STAI-state significantly mediated the association between maternal chronotype and breastfeeding duration (*β*_indirect_ = 0.0039 (0.0004, 0.009) and *β*_indirect_ = 0.004 (0.0004, 0.009), respectively). No significant mediation effects were seen for EPDS and STAI-trait scores in models 1 and 2 (i.e., all 95% CIs included the value zero). However, when not adjusted for potential covariates, we observed that STAI-trait significantly mediated the association between maternal chronotype and breastfeeding duration (*β*_indirect_ = 0.0057 (0.0016, 0.011)) (Table 4).

### 3.5. Maternal Mood Did Not Mediate the Association Between Maternal Sleep Duration and Breastfeeding Duration

All maternal mood variables (EPDS, STAI-state, and STAI-trait) were tested separately as potential mediators of the association between maternal night sleep duration and breastfeeding duration. None of the maternal mood variables significantly mediated this association (see Supplementary Table S2).

### 3.6. Additional Analysis

Sensitivity analyses were conducted by additionally adjusting for GDM status in the final mediation analyses. Significant mediation effects remained for STAI-state with regard to the association between maternal chronotype and breastfeeding duration.

## 4. Discussion

This study provides novel evidence that maternal chronotype, particularly morningness tendencies, is associated with longer breastfeeding duration, and that prenatal state-anxiety partially mediates this relationship. Specifically, mothers with morningness tendencies were significantly more likely to breastfeed for 3 to <6 months, 6 to 12 months, and ≥12 months, compared to <1 month. This association could be related to more consistent daily routines, better alignment with social and work schedules, or healthier lifestyle behaviors typically observed in morning-type individuals [46], which may facilitate sustained breastfeeding practices. Moreover, we found that the association between maternal chronotype and the duration of any breastfeeding was mediated by maternal prenatal state-anxiety. Mothers with morningness tendencies were more likely to have lower levels of maternal state-anxiety symptoms, and in turn, they were more likely to breastfeed longer. However, no significant associations were seen between maternal night sleep duration and breastfeeding duration, as well as mediation through maternal prenatal mood.

Much of the literature has focused on elucidating the role of maternal circadian rhythms on lactation incompetence in animals [47,48], as well as variability in human milk composition [49]. To the best of our knowledge, this is the first study to examine the association between maternal chronotype and breastfeeding duration and reported mediation by maternal anxiety symptoms. Evidence suggests that an evening chronotype can influence women to adopt unhealthy behaviors, such as night eating during pregnancy [50], but whether there is a link with breastfeeding behaviors that may affect the duration of breastfeeding is unknown. More research is needed to explore the relationship between maternal chronotype and breastfeeding outcomes, as well as the causal mechanisms that underlie this relationship.

Past evidence has separately reported associations between maternal chronotype and maternal mood [22,23,24], as well as between maternal mood and breastfeeding outcomes [7,11,12,13,14], thus implying that maternal mood may serve as an underlying mechanism through which maternal chronotype can influence breastfeeding outcomes. Our findings expand those from other studies that found a link between pregnant evening chronotypes and risk of developing postpartum depressive symptoms [22,23,24]. In our study, maternal mood was assessed during the second trimester (i.e., 26–28 weeks of gestation), unlike the third trimester or postpartum periods commonly assessed by previous studies [12,13,22,23,24]. Despite the differences in time of assessments, our study similarly showed that morningness tendencies in women were associated with lower mood symptom scores, albeit only with prenatal anxiety, but not with depressive symptoms. In contrast with prior studies that found a link between prenatal depression and shortened breastfeeding duration [12], we found instead that lower prenatal anxiety symptoms (not depressive symptoms) were associated with a longer breastfeeding duration. Hence, this implies that breastfeeding duration can be impacted by maternal anxiety symptoms as early as the second trimester. This is consistent with previous studies showing that higher prenatal anxiety is associated with shorter breastfeeding duration or earlier cessation of breastfeeding [7,11,12,13,14,16,17], highlighting the relevance of maternal mood during pregnancy for subsequent feeding behaviors.

In this study, maternal state-anxiety explained a very small, but significant part of the association between maternal chronotype and breastfeeding duration (*β*_indirect_ = 0.004). Although the mediation was statistically significant, the magnitude of the indirect effect was small, suggesting that its clinical relevance may be limited and should be interpreted with caution. These findings suggest a potential explanatory pathway, rather than confirming a behavioral or causal mechanism. As the study participants comprised mainly low-risk individuals who manifest non-clinical and subclinical mood symptoms [51], and given that only a small proportion of participants (5.3% of 340 participants) exhibited evening chronotype categorization (MEQ score: ≤41), it is possible that the low variability of mood symptoms and chronotype led to the observed small indirect effects. This explanation may also explain our null results seen with depressive symptoms. Additionally, prior studies often assessed maternal depressive symptoms in late pregnancy or postpartum periods [52], whereas we measured symptoms in mid-pregnancy, which may contribute to differences in findings. Similar null associations have been observed in other low-risk or non-clinical populations [53,54] like our sample population, suggesting that the influence of depressive symptoms on breastfeeding may be more pronounced in high-risk populations or when symptoms are clinically significant. Indeed, some previous studies have not found significant associations of both maternal depressive and anxiety symptoms with breastfeeding practices [7,11,12,13]. For example, a cohort study that examined the effect of maternal anxiety or depression on breastfeeding outcomes found a relationship between maternal anxiety and reduced breastfeeding, but not with maternal depression. The authors concurred that the lack of significance may be attributed to small sample sizes [14]. Whether low-risk or high-risk maternal mood symptoms represent behavioral pathways through which maternal chronotype affects breastfeeding outcomes remains to be investigated. Interestingly, a disparity in results exists between state- and trait-anxiety when tested for mediation. This is remarkable, as state- and trait-anxiety have been found to be commonly associated in our cohort sample [14,55]. This may suggest that with regard to breastfeeding outcomes, transient anxiety symptoms (i.e., state-anxiety) play a larger role compared to anxiety traits that are based on personality characteristics (i.e., trait-anxiety) [39]. Interestingly, Stuebe et al. (2019) reported that higher state-anxiety symptoms, but not trait-anxiety, during late pregnancy were associated with earlier use of infant formula or cessation of breastfeeding [17]. While our findings suggest that transient anxiety symptoms may play a larger role than stable trait-anxiety in influencing breastfeeding duration, other unmeasured factors could also contribute. These may include psychosocial stressors, maternal breastfeeding self-efficacy, and changes in maternal mood across the postpartum period, which were not captured in the present study. Future research should examine these variables to better elucidate the mechanisms underlying breastfeeding behaviors. Hence, it is useful for future studies to replicate this finding, as this may provide opportunities to intervene with regard to anxiety symptoms as one of the targets for promoting breastfeeding duration. Practical strategies such as relaxation techniques, breastfeeding support, and mindfulness-based practices have been associated with reduced anxiety and may be feasible for supporting breastfeeding outcomes in mothers with eveningness tendencies [56].

Although not significant, the trends from our individual regression results implied the possibility that longer maternal night sleep duration as early as the prenatal period (i.e., 26–28 weeks of gestation) may be associated with longer breastfeeding duration and better maternal prenatal mood scores. These findings parallel those from another study that found a link between reduced sleep duration and shorter breastfeeding duration during the postpartum period [33], as well as those from some studies that found a link between compromised sleep duration/quality and increased mood disturbances during the perinatal period [29]. More studies are needed to elucidate the effects of antenatal and postpartum maternal sleep on breastfeeding duration, given that biological or social factors during these periods may variably influence maternal sleep.

## 5. Strengths, Limitations, and Future Directions

This study has several strengths. Firstly, we adjusted for various known confounding factors in our analysis [5,8,9,10], including GDM status, which is known to influence breastfeeding success and is also associated with maternal chronotype [43,44]. Still, we acknowledge that other factors, such as breastfeeding intention and the working status of mothers (e.g., career, type or timing of jobs), may also influence breastfeeding [57]. One study, however, found that working rotating night shifts may not affect breast milk volume in employed breastfeeding mothers who use the breastfeeding room during working hours [58]. In our study, the majority of women held full-time employment during early pregnancy (*N* = 230, 67.6% of 340 participants) and worked 41.2 ± 10.4 h in total during an average week. Only 6.1% out of *N* = 230 women worked night shifts, and most of the women reported an intention to breastfeed (*N* = 324, 97.0% of 334 participants). Hence, there was not much variation in these measures to be adjusted for in the analysis. Furthermore, some studies did not find a link between maternal chronotype or mood and breastfeeding intention [12,59]. Secondly, to the best of our knowledge, our study is the first to show that mothers with morningness tendencies were more likely to breastfeed longer, and that this was mediated via lower levels of anxiety during the mid-pregnancy period. However, we did not investigate this further in terms of breastfeeding exclusively. The reason is that full breastfeeding rates were low in our cohort study [5] and there may be no actual variation in duration of exclusive/full breastfeeding to assess dose–response relationships with maternal chronotype. It would be useful to understand whether maternal chronotype and maternal mood influence exclusive or full breastfeeding, given that exclusive breastfeeding is recommended for the first 6 months after birth [4]. Further, our study extends the previous literature that reported the link between higher maternal prenatal anxiety symptoms (typically state-anxiety) during the mid- and/or late-pregnancy periods and poor breastfeeding outcomes [16,17].

This study has several methodological limitations. The mediation analyses reflect statistical associations rather than confirmed behavioral mechanisms or causal pathways, and the modest indirect mediation effects should be interpreted with caution. Key variables, including maternal chronotype, mid-pregnancy mood, and breastfeeding duration, were assessed via self-report, which may introduce measurement bias. Maternal chronotype was measured at 54 months postpartum, which may limit temporal validity. However, chronotype is considered a relatively stable trait in adults [26], and it has been shown that changes in maternal chronotype returned to pre-pregnancy levels by the third trimester [27,28]. Nevertheless, future studies should ideally assess maternal chronotype at mid-pregnancy or at the time of breastfeeding duration assessment to improve temporal validity and causal interpretation. Maternal mood was assessed only once during mid-pregnancy (26–28 weeks of gestation), which may not fully capture antenatal or postpartum mood trajectories, and state- and trait-anxiety can vary over time. Given that a large proportion of our study sample had already ceased breastfeeding by 3–6 months postpartum [5], we were unable to include an additional assessment of maternal mood (STAI, EPDS) during this period to strengthen the mediation analyses and better capture postpartum mood changes that may influence breastfeeding outcomes. Also, recent evidence has shown that maternal depression and anxiety levels may change across pregnancy trimesters [60]. While state-anxiety examines current symptoms that can vary dynamically with time, trait-anxiety, on the other hand, is more stable and persistent [39]. Further investigations should evaluate whether differences in trajectories of change in maternal chronotype and mood across the peripartum period can influence breastfeeding outcomes. Chronotype not only affects performance during the day [61], but also affects sleep [25], and breastfeeding practices can also influence sleep [62], which may explain the spurious associations seen from our null results with maternal night sleep duration. Maternal night sleep duration was measured using a single self-reported item, which may have limited precision. Although breastfeeding duration was collected in categories, it was treated as a continuous variable in our analyses; this approach assumes approximately linear relationships and equal spacing between categories, which may slightly affect model assumptions, and future studies could consider ordinal regression or survival analysis for more precise modeling. In addition, we used a complete-case analysis, which may introduce selection bias, as included participants differed from excluded participants in parity and maternal age; these variables were also included as covariates in the adjusted analyses. Future studies could use approaches such as multiple imputation to handle missing data and reduce potential bias. More research considering such influential factors is needed when discerning the true relationship between maternal sleep, maternal mood, and breastfeeding practices. Additionally, simple contextual covariates such as family support, access to breastfeeding advice, or timing of return to work could be collected and adjusted for in models to better account for social and environmental influences on breastfeeding. Finally, the participants in our cohort were primarily low-risk, highly motivated mothers with the intention to breastfeed, and thus, the findings may not be generalizable to populations with higher risk profiles, lower breastfeeding motivation, or different sociodemographic characteristics. However, our study participants were recruited from the two largest maternity hospitals in Singapore. Future studies should aim to address these limitations using objective, repeated, and validated measures in larger and more diverse populations.

## 6. Conclusions

In conclusion, the results of this study suggest that maternal state-anxiety may be a potential behavioral pathway through which maternal chronotype is associated with breastfeeding duration. These findings indicate that maternal anxiety during pregnancy, particularly among women with eveningness tendencies, may be relevant to breastfeeding behaviors. Further longitudinal and interventional studies are needed to determine whether targeting maternal mood can effectively promote breastfeeding duration.

## Figures and Tables

**Table 1:** Comparison of infant and maternal characteristics with duration of any breastfeeding.

		Duration of Any Breastfeeding (*N* = 340)					
Characteristics	Mean ± SD/ *N* (%)	<1 Month (*N* = 68)	1 to <3 Months (*N* = 64)	3 to <6 Months (*N* = 59)	6 to 12 Months (*N* = 71)	≥12 Months (*N* = 78)	*p* ^c^
Infant							
Male	171 (50.3)	33 (48.5)	27 (42.2)	35 (59.3)	31 (43.7)	45 (57.7)	0.156
Birth weight, kg	3.2 ± 0.4	3.1 ± 0.4	3.2 ± 0.4	3.2 ± 0.4	3.2 ± 0.3	3.2 ± 0.3	0.313
Gestational age, week	39.1 ± 1.0	38.9 ± 1.0	39.1 ± 1.0	39.2 ± 1.1	39.2 ± 1.0	39.1 ± 1.0	0.615
Maternal							
Maternal age, year	31.1 ± 5.1	29.8 ± 6.0	30.7 ± 5.3	32.4 ± 4.6	30.8 ± 4.7	32.0 ± 4.4	0.021 *
Maternal ethnicity							
Chinese	202 (59.4)	38 (55.9)	27 (42.2)	35 (59.3)	51 (71.8)	51 (65.4)	0.011 *
Malay	89 (26.2)	22 (32.4)	27 (42.2)	16 (27.1)	9 (12.7)	15 (19.2)	
Indian	49 (14.4)	8 (11.7)	10 (15.6)	8 (13.6)	11 (15.5)	12 (15.4)	
Pre-pregnancy BMI, kg/m^2^	22.6 ± 4.4	22.9 ± 5.0	24.8 ± 5.8	22.8 ± 3.6	22.0 ± 3.3	21.2 ± 3.0	<0.001 ***
Missing data	32 (9.4)	11 (16.2)	4 (6.3)	3 (5.1)	9 (12.7)	5 (6.4)	
Parity							
0	160 (47.0)	27 (39.7)	32 (50.0)	26 (44.1)	37 (52.1)	38 (48.7)	0.495
1	92 (27.1)	16 (23.5)	20 (31.3)	16 (27.1)	18 (25.4)	22 (28.2)	
≥2	88 (25.9)	25 (36.8)	12 (18.7)	17 (28.8)	16 (22.5)	18 (23.1)	
GDM							
Yes	56 (16.5)	10 (14.7)	12 (18.8)	10 (16.9)	10 (14.1)	14 (17.9)	0.939
No	271 (79.7)	54 (79.4)	48 (75.0)	48 (81.4)	58 (81.7)	63 (80.8)	
Missing data	13 (3.8)	4 (5.9)	4 (6.2)	1 (1.7)	3 (4.2)	1 (1.3)	
Maternal education							
<Postsecondary	95 (27.9)	41 (60.3)	21 (32.8)	13 (22.0)	12 (16.9)	8 (10.3)	<0.001 ***
Postsecondary	114 (33.5)	18 (26.5)	30 (46.9)	26 (44.1)	19 (26.8)	21 (26.9)	
≥University	126 (37.1)	8 (11.8)	12 (18.8)	19 (32.2)	39 (54.9)	48 (61.5)	
Missing data	5 (1.5)	1 (1.4)	1 (1.5)	1 (1.7)	1 (1.4)	1 (1.3)	
Household income, SGD							
$0–$1999	40 (11.8)	18 (26.5)	10 (15.6)	5 (8.5)	5 (7.0)	2 (2.6)	<0.001 ***
$2000–$3999	101 (29.7)	26 (38.2)	26 (40.6)	18 (30.5)	14 (19.7)	17 (21.8)	
≥$4000	181 (53.2)	19 (27.9)	25 (39.1)	33 (55.9)	48 (67.6)	56 (71.8)	
Missing data	18 (5.3)	5 (7.4)	3 (4.7)	3 (5.1)	4 (5.7)	3 (3.8)	
Mode of delivery							
Vaginal delivery	244 (71.8)	45 (66.2)	45 (70.3)	36 (61.0)	55 (77.5)	63 (80.8)	0.089
Intrapartum caesarean section	50 (14.7)	14 (20.6)	8 (12.5)	13 (22.0)	5 (7.0)	10 (12.8)	
Non-labor caesarean section	46 (13.5)	9 (13.2)	11 (17.2)	10 (17.0)	11 (15.5)	5 (6.4)	
Maternal chronotype ^a^							
MEQ score	55.1 ± 8.3	52.1 ± 8.2	54.4 ± 8.4	56.4 ± 7.4	57.0 ± 7.7	55.9 ± 8.9	0.004 **
Maternal night sleep duration	7.0 ± 1.4	7.0 ± 1.4	6.8 ± 1.8	7.0 ± 1.1	7.2 ± 1.4	7.1 ± 1.4	0.771
Maternal mood ^b^							
EPDS score	7.8 ± 4.5	9.1 ± 5.1	8.1 ± 3.8	7.5 ± 4.8	6.8 ± 4.1	7.7 ± 4.4	0.052
STAI-state score	34.9 ± 9.9	39.4 ± 10.2	36.0 ± 9.4	34.4 ± 10.5	32.3 ± 7.9	32.6 ± 10.1	<0.001 ***
STAI-trait score	36.6 ± 8.7	40.5 ± 9.0	37.4 ± 8.1	35.6 ± 9.0	34.5 ± 7.7	35.2 ± 8.5	<0.001 ***

Values are *N*, *N* (%), and means ± SDs unless otherwise indicated. BMI, Body Mass Index; EPDS, Edinburgh Postnatal Depression Scale; GDM, gestational diabetes mellitus; MEQ, Morningness–Eveningness Questionnaire; SGD, Singapore Dollars, STAI, State–Trait Anxiety Inventory. ^a^ Maternal chronotype questionnaire administered at 54 months postpartum. Post hoc reference category <1 month: 3 to <6 months (*p* = 0.012); 6 to 12 months (*p* = 0.002); and ≥12 months (*p* = 0.019). ^b^ Maternal mood questionnaires administered at 26–28 weeks of gestation. ^c^ *p*-values derived from Chi-square tests for categorical variables and one-way analysis of variance (ANOVA) tests (overall between groups) for continuous variables. * *p* ≤ 0.05, ** *p* ≤ 0.01, *** *p* ≤ 0.001 were statistically significant.

**Table 2:** Multivariable linear regression of maternal chronotype (MEQ score), maternal sleep duration, maternal mood (EPDS, STAI-state and STAI-trait scores), and duration of any breastfeeding.

	Duration of Any Breastfeeding (Months)					
	Unadjusted		Adjusted Model 1		Adjusted Model 2	
	*N*		*N*		*N*	
MEQ score	340	0.03 (0.01, 0.05) **	308	0.02 (0.0005, 0.04) **	308	0.02 (0.001, 0.04) **
Night sleep duration, h	229	0.06 (−0.07, 0.20)	211	0.01 (−0.12, 0.13)	211	0.01 (−0.12, 0.13)
EPDS score	340	−0.04 (−0.08, −0.01) **	308	−0.01 (−0.05, 0.02)	308	−0.01 (−0.05, 0.02)
STAI-state score	340	−0.04 (−0.05, −0.02) **	308	−0.02 (−0.04, −0.01) **	308	−0.02 (−0.04, −0.01) **
STAI-trait score	340	−0.04 (−0.06, −0.02) **	308	−0.02 (−0.04, −0.004) **	308	−0.02 (−0.04, −0.004) **

Values are unstandardized beta coefficients (95% CIs). Adjusted model 1 was adjusted for maternal age, maternal ethnicity, maternal education, parity, and pre-pregnancy BMI. Adjusted model 2 was adjusted for maternal age, maternal ethnicity, maternal education, parity, pre-pregnancy BMI, and mode of delivery. EPDS, Edinburgh Postnatal Depression Scale; MEQ, Morningness–Eveningness Questionnaire; STAI, State–Trait Anxiety Inventory. ** *p* ≤ 0.01 were statistically significant.

**Table 3:** Multivariable linear regression of maternal chronotype (MEQ score), maternal sleep duration, and maternal mood (EPDS, STAI-state, STAI-trait scores).

	Maternal Mood		
	EPDS Score	STAI-State Score	STAI-Trait Score
MEQ score			
Unadjusted (*N* = 340)	−0.05 (−0.10, 0.01)	−0.18 (−0.31, −0.06) *	−0.17 (−0.28, −0.06) *
Adjusted model 1 (*N* = 308)	−0.04 (−0.10, 0.02)	−0.19 (−0.32, −0.06) *	−0.17 (−0.29, −0.06) *
Adjusted model 2 (*N* = 308)	−0.04 (−0.10, 0.02)	−0.19 (−0.32, −0.06) *	−0.18 (−0.29, −0.06) *
Night sleep duration, h			
Unadjusted (*N* = 229)	−0.15 (−0.56, 0.25)	−0.14 (−1.09, 0.81)	−0.32 (−1.13, 0.50)
Adjusted model 1 (*N* = 211)	−0.08 (−0.49, 0.34)	−0.02 (−0.96, 0.92)	−0.24 (−1.07, 0.59)
Adjusted model 2 (*N* = 211)	−0.08 (−0.49, 0.34)	−0.02 (−0.96, 0.92)	−0.24 (−1.07, 0.60)

Values are unstandardized beta coefficients (95% CIs). Adjusted model 1 was adjusted for maternal age, maternal ethnicity, maternal education, parity, and pre-pregnancy BMI. Adjusted model 2 was adjusted for maternal age, maternal ethnicity, maternal education, parity, pre-pregnancy BMI, and mode of delivery. EPDS, Edinburgh Postnatal Depression Scale; MEQ, Morningness–Eveningness Questionnaire; STAI, State–Trait Anxiety Inventory. * *p* ≤ 0.05 is statistically significant.

**Table 4:** Mediation models between maternal chronotype (MEQ score), maternal mood (EPDS, STAI-state, STAI-trait scores), and duration of any breastfeeding.

		Mediation Coefficients		
	*N*	c	c’	a × b
EPDS score				
Unadjusted	340	0.03 (0.01, 0.05) *	0.03 (0.01, 0.05) *	0.002 (−0.0004, 0.005)
Adjusted model 1	308	0.02 (0.0005, 0.04) *	0.02 (0.00003, 0.04) *	0.0004 (−0.001, 0.003)
Adjusted model 2	308	0.02 (0.001, 0.04) *	0.02 (0.0007, 0.04) *	0.0003 (−0.001, 0.002)
STAI-state score				
Unadjusted	340	0.03 (0.01, 0.05) *	0.02 (0.006, 0.04) *	0.006 (0.002, 0.01) *
Adjusted model 1	308	0.02 (0.0005, 0.04) *	0.01 (−0.004, 0.03)	0.0039 (0.0004, 0.009) *
Adjusted model 2	308	0.02 (0.001, 0.04) *	0.02 (−0.003, 0.03)	0.004 (0.0004, 0.009) *
STAI-trait score				
Unadjusted	340	0.03 (0.01, 0.05) *	0.02 (0.006, 0.04) *	0.006 (0.002, 0.01) *
Adjusted model 1	308	0.02 (0.0005, 0.04) *	0.02 (−0.003, 0.03)	0.003 (−0.00004, 0.008)
Adjusted model 2	308	0.02 (0.001, 0.04) *	0.02 (−0.002, 0.03)	0.003 (−0.00005, 0.008)

Values are unstandardized beta coefficients (95% CIs) with 10,000 bootstrap samples for 95% bootstrap CIs. Adjusted model 1 was adjusted for maternal age, maternal ethnicity, maternal education, parity, and pre-pregnancy BMI. Adjusted model 2 was adjusted for maternal age, maternal ethnicity, maternal education, parity, pre-pregnancy BMI, and mode of delivery. c: association between maternal chronotype and breastfeeding duration excluding maternal mood (total effect); c’: association between maternal chronotype and breastfeeding duration controlled for maternal mood (direct effect); a × b: mediation effect or indirect effect through which maternal chronotype influences breastfeeding duration (product of a and b). EPDS, Edinburgh Postnatal Depression Scale; MEQ, Morningness–Eveningness Questionnaire; STAI, State–Trait Anxiety Inventory. * *p* ≤ 0.05 is considered significant.

## Data Availability

The data presented in this study are available on request from the corresponding author due to reasons of sensitivity.

## Supplementary Materials

The following supporting information can be downloaded at: https://www.mdpi.com/article/10.3390/nu18030405/s1, Table S1: Comparison of participant characteristics between those participants that are included and not included in the final analyses, Table S2: Mediation models between maternal night sleep duration, maternal mood (EPDS, STAI-state, STAI-trait scores), and duration of any breastfeeding.

## Abbreviations

The following abbreviations are used in this manuscript:

DAG	Directed Acyclic Graph
MEQ	Horne–Östberg Morningness–Eveningness Questionnaire
PSQI	Pittsburgh Sleep Quality Index
EPDS	Edinburgh Postnatal Depression Scale
SGD	Singapore Dollars
STAI	State–Trait Anxiety Inventory
GUSTO	Growing Up in Singapore Towards healthy Outcomes
GDM	Gestational Diabetes Mellitus
OGTT	Oral Glucose Tolerance Test
CI	Confidence Intervals
VIF	Variance Inflation Factor
BMI	Body Mass Index
