# Supplementary material for: Mid-Pregnancy Maternal Anxiety Mediates the Association Between Maternal Chronotype and Breastfeeding Duration
Source: Nutrients. 2026 Jan 26;18(3):405. doi: 10.3390/nu18030405 (PMC12899007; doi:10.3390/nu18030405)
Supplement: Supplementary file 1 [file nutrients-18-00405-s001.zip › nutrients-4088090-supplementary.pdf]

Supplementary

Table S1. Comparison of participant characteristics between those participants that are included and not included in the final analyses (n = 966).

	Participants included (n = 340)	Participants not included (n = 626)	p^{a}
	Mean ± SD/n (%)	Mean ± SD/n (%)	
Infant			
Child's sex			0.251
Male	171 (50.3)	340 (54.3)	
Birth weight, kg	3.2 ± 0.4	3.2 ± 0.4	0.899
Gestational age, wk	39.1 ± 1.0	39.1 ± 1.0	0.933
Maternal			
Maternal age, y	31.1 ± 5.1	30.2 ± 5.0	0.009**
Maternal ethnicity			0.071
Chinese	202 (59.4)	336 (53.7)	
Malay	89 (26.2)	164 (26.2)	
Indian	49 (14.4)	126 (20.1)	
Pre-pregnancy BMI, kg/m^2	22.6 ± 4.4	22.8 ± 4.5	0.588
Missing data	32 (9.4)	58 (9.3)	
Parity			<0.001***
0	160 (47.0)	256 (40.9)	
1	92 (27.1)	252 (40.3)	
≥2	88 (25.9)	118 (18.8)	
GDM			0.927
Yes	56 (16.5)	101 (16.1)	
No	271 (79.7)	497 (79.4)	
Unclassifiable/Missing data	13 (3.8)	28 (4.5)	
Maternal education			0.378
<Postsecondary	95 (27.9)	193 (30.8)	
Postsecondary	114 (33.5)	219 (35.0)	

≥University	126 (37.1)	205 (32.8)	
Missing data	5 (1.5)	9 (1.4)	
Household income, Singapore dollars			0.408
$0-$1999	40 (11.8)	89 (14.2)	
$2000-$3999	101 (29.7)	184 (29.4)	
≥$4000	181 (53.2)	304 (48.6)	
Missing data	18 (5.3)	49 (7.8)	
Mode of delivery			0.963
Vaginal delivery	244 (71.8)	451 (72.0)	
Intrapartum caesarean section	50 (14.7)	94 (15.0)	
Non-labour caesarean section	46 (13.5)	81 (13.0)	

[a]*P* values are based on Pearson's chi-square tests or two-sided Fisher's exact test for categorical variables, and Mann-Whitney U test for continuous variables. BMI, Body Mass Index; EPDS, Edinburgh Postnatal Depression Scale; GDM, gestational diabetes mellitus; MEQ, Morningness-Eveningness Questionnaire; STAI, State-Trait Anxiety Inventory. $P < .05$ values are significant.
Note: Participants that are included in the final analyses had all MEQ, EPDS, STAI-state, STAI-trait, and breastfeeding duration data while those excluded from the analyses did not have either MEQ, EPDS, STAI-state, STAI-trait, and/or breastfeeding duration data.
$^{**}P \leq 0.01$, $^{***}P \leq 0.001$ were statistically significant.

Table S2. Mediation models between maternal night sleep duration, maternal mood (EPDS, STAI-state, STAI-trait scores), and duration of any breastfeeding.

		**Mediation coefficients**		
	N	**c**	**c'**	**a x b**
EPDS score				
Unadjusted	229	0.06 (-0.07, 0.20)	0.06 (-0.08, 0.19)	0.006 (-0.01, 0.03)
Adjusted model 1	211	0.005 (-0.12, 0.13)	0.004 (-0.12, 0.13)	0.0009 (-0.01, 0.01)
Adjusted model 2	211	0.005 (-0.12, 0.13)	0.004 (-0.12, 0.13)	0.0008 (-0.01, 0.01)
STAI-state score				
Unadjusted	229	0.06 (-0.07, 0.20)	0.06 (-0.07, 0.19)	0.006 (-0.04, 0.05)
Adjusted model 1	211	0.01 (-0.12, 0.13)	0.005 (-0.12, 0.13)	0.0006 (-0.04, 0.03)
Adjusted model 2	211	0.005 (-0.12, 0.13)	0.004 (-0.12, 0.13)	0.0006 (-0.04, 0.03)
STAI-trait score				
Unadjusted	229	0.06 (-0.07, 0.20)	0.05 (-0.08, 0.18)	0.01 (-0.03, 0.05)
Adjusted model 1	211	0.005 (-0.12, 0.13)	-0.001 (-0.12, 0.12)	0.007 (-0.03, 0.03)
Adjusted model 2	211	0.005 (-0.12, 0.13)	-0.002 (-0.12, 0.12)	0.006 (-0.02, 0.03)

Values are unstandardized beta coefficients (95% CIs) with 10,000 bootstrap samples for 95% bootstrap CIs. Adjusted model 1 were adjusted for maternal age, maternal ethnicity, maternal education, parity, and pre-pregnancy BMI. Adjusted model 2 were adjusted for maternal age, maternal ethnicity, maternal education, parity, pre-pregnancy BMI, and mode of delivery.

c: association between maternal night sleep and breastfeeding duration excluding maternal mood (total effect); c': association between maternal night sleep and breastfeeding duration controlled for maternal mood (direct effect); a x b: mediation effect or indirect effect through which maternal night sleep influences breastfeeding duration (product of a and b). EPDS, Edinburgh Postnatal Depression Scale; STAI, State-Trait Anxiety Inventory.
